# A Sustained-Release Butyrate Tablet Suppresses Ex Vivo T Helper Cell Activation of Osteoarthritis Patients in a Double-Blind Placebo-Controlled Randomized Trial

**DOI:** 10.3390/nu16193384

**Published:** 2024-10-04

**Authors:** Sandra G. P. J. Korsten, Merel Hartog, Alinda J. Berends, Marije I. Koenders, Calin D. Popa, Herman Vromans, Johan Garssen, Cornelia H. M. van de Ende, Jan P. W. Vermeiden, Linette E. M. Willemsen

**Affiliations:** 1 Division of Pharmacology, Utrecht Institute for Pharmaceutical Sciences, Utrecht University, 3584 CG Utrecht, The Netherlandsj.garssen@uu.nl (J.G.); 2 Tiofarma B.V., 3261 ME Oud-Beijerland, The Netherlands; 3 Department of Research, Sint Maartenskliniek, 6574 NA Ubbergen, The Netherlands; m.hartog@maartenskliniek.nl (M.H.); e.vandenende@maartenskliniek.nl (C.H.M.v.d.E.); 4 Department of Rheumatology, Sint Maartenskliniek, 6574 NA Ubbergen, The Netherlands; c.popa@maartenskliniek.nl; 5 Department of Rheumatology, Radboud University Medical Center, 6525 GA Nijmegen, The Netherlands; 6 Division of Pharmaceutics, Utrecht Institute for Pharmaceutical Sciences, Utrecht University, 3584 CG Utrecht, The Netherlands; 7 Danone/Nutricia Research B.V., 3584 CT Utrecht, The Netherlands; 8 Birr Beheer B.V., 3633 AG Vreeland, The Netherlands

**Keywords:** butyrate, short-chain fatty acid, osteoarthritis, non-communicable diseases, low-grade inflammation, intestinal barrier

## Abstract

Degenerative joint disease osteoarthritis (OA) is characterized by the degeneration of cartilage, synovial inflammation and low-grade systemic inflammation in association with microbial dysbiosis and intestinal barrier defects. Butyrate is known for its anti-inflammatory and barrier protective effects and might benefit OA patients. In a double-blind placebo-controlled randomized trial, the effects of four to five weeks of oral treatment with sustained-release (SR) butyrate tablets (600 mg/day) on systemic inflammation and immune function were studied in hand OA patients. Serum markers for systemic inflammation and lipopolysaccharide (LPS) leakage were measured and ex vivo stimulation of whole blood or peripheral blood mononuclear cells (PBMCs) was performed at baseline and after treatment. Butyrate treatment did not affect the serum markers nor the cytokine release of ex vivo LPS-stimulated whole blood or PBMCs nor the phenotype of restimulated monocytes. By contrast, butyrate treatment reduced the percentage of activated T helper (Th) cells and the Th17/Treg ratio in αCD3/CD28-activated PBMCs, though cytokine release upon stimulation remained unaffected. Nevertheless, the percentage of CD4+IL9+ cells was reduced by butyrate as compared to the placebo. In both groups, the frequency of Th1, Treg, Th17, activated Th17, CD4+IFNγ+ and CD4+TNFα+ cells was reduced. This study shows a proof of principle of some immunomodulatory effects using a SR butyrate treatment in hand OA patients. The inflammatory phenotype of Th cells was reduced, as indicated by a reduced percentage of Th9 cells, activated Th cells and improved Th17/Treg balance in ex vivo αCD3/CD28-activated PBMCs. Future studies are warranted to further optimize the butyrate dose regime to ameliorate inflammation in OA patients.

## 1. Introduction

Osteoarthritis (OA) is a degenerative joint disease with rising prevalence [1]. OA can be classified as a non-communicable disease and is characterized by degeneration of cartilage, synovial inflammation and low-grade systemic inflammation [2]. Clinical evidence shows that this inflammation is associated with microbial dysbiosis and intestinal barrier defects [1,3,4,5,6,7]. It has been hypothesized that dysbiosis of the microbiome leads to local inflammation in the gut and increased intestinal permeability, contributing to an influx of bacterial components such as lipopolysaccharides (LPSs) into the bloodstream, resulting in systemic low-grade inflammation [8,9]. This systemic inflammation might attribute to the development of OA, resulting in joint pain [10,11,12,13,14,15]. Furthermore, the inflammation might cause a positive feedback loop by maintaining the intestinal barrier defect, thereby facilitating a continuous influx of LPSs and consequently sustaining inflammation and, in this way, driving the pathology of OA. Markers for systemic LPS leakage are, amongst others, LPS-IgG and LPS-binding protein (LBP). A rise in serum LBP is associated with increased knee OA progression [9]. Additionally, the microbiome is known for its capacity to produce biologically active immunomodulatory molecules such as short-chain fatty acids (SCFAs) via the fermentation of fibers [1,4,10]. Dysbiosis of the microbiome might lead to altered production of SCFAs, although the latter has not been confirmed in OA patients yet [16,17,18].

In the joints of OA patients, activated cells of the innate and adaptive immune system are found, and the synovial fluid contains increased concentrations of inflammatory mediators which can affect homeostasis in the cartilage [19,20,21]. In particular, activated monocytes/macrophages and T helper (Th)1, Th17 and Th9 cells play a role in the pathophysiology of OA [22,23], with LPSs being a main driver of macrophage activation. Increased levels of IL-17a, IL-9, and Th17 and Th9 cells can be observed in the circulation of OA patients and are associated with disease activity [23,24,25,26]. In addition, regulatory T-cell (Treg) function and Treg/Th17 balance may be disturbed [25,27].

Currently, no disease-modifying treatment is available for OA patients and the current standard therapy consists of patient education, exercise therapy and pain medication [28,29]. The SCFA butyrate is known for its anti-inflammatory and barrier-improving properties and could therefore be a possible treatment for patients with OA [30,31,32], in particular when considering intestinal dysbiosis, which might cause butyrate shortage in the intestine of these patients [16,17,18].

The direct effects of butyrate on peripheral blood mononuclear cells (PBMCs) or innate immune cells like monocytes, which are precursors for tissue-resident macrophages, have previously been studied. In these studies, butyrate was shown to reduce the release of various pro-inflammatory cytokines by activated PBMCs and to modulate immune cell phenotypes [33,34,35,36,37,38,39,40,41]. In addition, butyrate can improve the intestinal epithelial barrier, protect against inflammatory-mediated barrier disruption and suppress the activation of epithelial cells in vitro [36,42,43,44,45]. It should be realized that in these models there was direct and constant contact of butyrate with the cells. So far, it is unknown whether oral pharmacotherapy with butyrate can establish an anti-inflammatory response in human extraintestinal pathologies associated with intestinal barrier defects, such as that in patients with OA.

The aim of this study was to investigate the anti-inflammatory properties of sustained-release butyrate tablets in patients with hand OA within a double-blind placebo-controlled randomized trial. The sustained-release butyrate tablet was developed to release sufficient amounts of butyrate that, in theory, should be able to achieve pharmacologically active concentrations along the small intestine [46]. We hypothesized that this dosage form and dose of butyrate would have beneficial effects on the intestinal barrier and, via this way, on the influx of LPSs (as indicated by serum LPS IgG and LBP levels) and systemic inflammation, which would affect the inflammatory potential of monocytes and Th cells and the Th17/Treg cell balance as well. In this manuscript, we outline the effect of the sustained-release butyrate tablet on in vivo and ex vivo immune parameters.

## 2. Materials and Methods

### 2.1. Participants

Thirty-three patients (27 females and 6 males, age 50 to 74 years) participated in this study after giving their informed consent. The participants were randomly allocated to either the placebo (*n* = 17; 13 females, 4 males; 13 with disease duration of ≥5 years) or the sustained-release butyrate tablet group (*n* = 16; 14 females, 2 males; 8 with disease duration of ≥5 years) (Table 1). This clinical study was approved by the Ethical Committee of the Radboud University Medical Center (Nijmegen, The Netherlands, protocol number: NL73382.091.21, approval date: 3 February 2022) and was conducted in full accordance with the principles of the Declaration of Helsinki. The clinical trial was registered in the European Union Clinical Trials Register with reference code 2020-001071-33 and conducted at the Sint Maartenskliniek (Ubbergen, The Netherlands). To be eligible for inclusion in the study, participants had to be ≥50 and ≤80 years of age, have a Body Mass Index (BMI) > 20 and <30 kg/m^2^ and have hand OA, according to the 1990 American College of Rheumatology (ACR) diagnostic criteria for hand OA, in both hands. Pain scored by the numeric pain rating scale (NRS) during hand activity needed to be ≥4 and ≤8 (scale 0–10), during 15 of the last 30 days. Exclusion criteria were the use of antibiotics within three months before the start of the study, use of NSAIDs, use of immunosuppressants, previous surgery of one of the hands, and a cerebro- or cardiovascular incident within 6 months before the start of the study. Further exclusion criteria were diabetes or other chronic inflammatory diseases or autoimmune diseases, cognitive deficits affecting the scoring process, fibromyalgia or any other syndrome or condition that could interfere with the assessment of pain. In addition, severe current psychiatric disorders assessed by a physician, self-reported consumption of >2 units of alcohol per day, intramuscular or intraarticular corticosteroid injections within four weeks before the start of the study, an estimated glomerular filtration rate (eGFR) < 30 mL/min/1.73 m^2^ and alanine aminotransferase (ALAT) < 1.5 ULN were exclusion criteria.

### 2.2. Design of the Double-Blind Placebo-Controlled Randomized Clinical Trial

**Figure 1:**
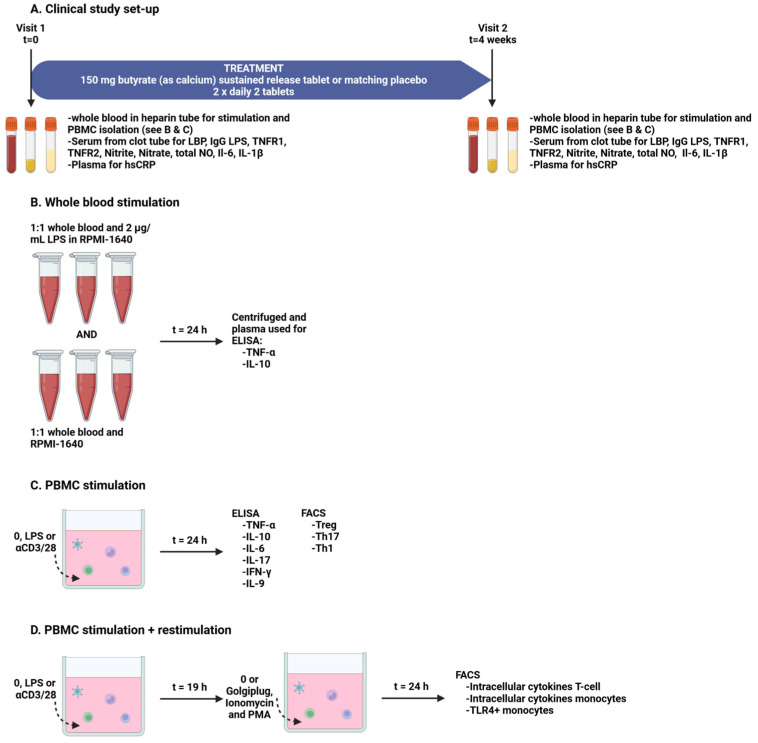
Clinical study set-up (**A**) and set-up of the methods used to stimulate and analyze the whole blood (**B**) and peripheral blood mononuclear cells (**C**,**D**). ELISA: enzyme-linked immunosorbent assay, hsCRP: highly sensitive C-reactive protein, IFN-γ: interferon-gamma, IL: interleukin, LPS: lipopolysaccharides, LBP: LPS-binding protein, NO: nitric oxide, PBMC: peripheral blood mononuclear cell, PMA: phorbol myristate acetate, Th: T helper cell, TNF-α: Tumor Necrosis Factor alpha, TNFR: TNF receptor, Treg: regulatory T-cell.

The patients participated in a double-blind randomized placebo-controlled study and were randomly allocated to either 150 mg butyrate (as calcium) sustained-release tablets (Tiofarma B.V., Oud-Beijerland, The Netherlands) or matching placebo tablets (see for composition the description of the tablet core below) (Tiofarma B.V.). Twice a day, two tablets (thus 600 mg butyrate/day in total) were taken for approximately four weeks (26–35 days, based on the availability of the patient and investigators for blood withdrawal and isolation of immune cells). Excipients used in the tablet core were hydroxypropyl methylcellulose (74 mg), silicified microcrystalline cellulose (99 mg) and magnesium stearate, and the tablets were coated with a taste-masking coating consisting of talc, titanium dioxide, polyethylene glycol 6000, simethicone emulsion, Eudragit RL 30 D and triethyl citrate. The tablet was formulated to release >0.08 mmol of butyrate per h within the time frame of 2 to 4 h post-ingestion, corresponding with the time frame the tablet is present in the small intestine [46]. Whole blood was collected in 4 × 10 mL heparin tubes, 1 × 3 mL heparin gel tube and 1 × 10 mL clot tube at the beginning (visit 1) and at the end of the study (visit 2) (Figure 1A). The blood in the 3 mL heparin gel tube was used to measure plasma levels of highly sensitive C-reactive protein (hsCRP). The clot tubes were spun down and serum was stored in cryovials at −80 °C until further basal serum measurements. The blood in the 10 mL heparin tubes was used for two follow up experiments. First, the whole blood was stimulated with LPS for 24 h and IL-10 and TNF-α were measured in the blood plasma. Second, PBMCs were isolated from the whole heparin blood and used for experiments in which the PBMCs were stimulated to measure cytokine release and to identify immune cell phenotypes (see experimental scheme Figure 1B–D). The effects on clinical parameters and the intestinal microbiome will be presented in another manuscript.

### 2.3. Basal Serum and Plasma hsCRP Measurements

Serum was collected at baseline (visit 1) and end of the study (visit 2) to measure LPS-binding protein (LBP), LPS IgG, soluble TNF-receptor 1 (sTNFR1), soluble TNF-receptor 2 (sTNFR2), nitrite, nitrate, total nitric oxide (NO), IL-6 and IL-1β. LBP (Thermo Fisher Scientific, Waltham, MA, USA), IgG LPS (Hycult Biotech, Uden, The Netherlands) and sCD14 (R&D systems (Minneapolis, MN, USA) were measured using an enzyme-linked immunosorbent assay (ELISA) according to the manufacturers instructions. Optical density was measured using a CLARIOstar Microplate Reader (BMG Labtech, Ortenberg, Germany). IL-6, IL-1β (Millipore, Milliplex human bone magnetic bead panel), sTNFR1 and sTNFR2 (Millipore, Milliplex human soluble cytokine receptor magnetic bead panel) were measured using an Antibody-Immobilized Beads immunoassay according to the manufacturers instruction and analyzed using a Bio-Plex 200 (Bio-Rad, Hercules, CA, USA). hsCRP was determined using the chemical analyzer Olympus type AU400.

### 2.4. Whole Blood Stimulation

On the same day as the whole heparin blood samples were drawn from the patients, whole blood samples were diluted 1:1 with plain RPMI-1640 (Sigma-Aldrich, St. Louis, MO, USA) or 2 μg/mL LPS (#tlrl-3pelps, Invivogen, Toulouse, France) in RPMI-1640 in triplo in an autoclaved screw cap micro tube (Sarstedt, Nümbrecht, Germany). Each tube was closed and gently mixed; thereafter, the cap was unscrewed a quarter turn to make sure the tubes were not completely closed. Tubes were incubated for 24 h in an incubator at 37 °C and 5% CO_2_ (Figure 1B). After 24 h of stimulation, the tubes were centrifuged for 10 min at 150× *g* at 20 °C followed by 10 min at 700× *g* at 20 °C in an Eppendorf centrifuge 5424 R. Blood plasma was transferred to a clean tube and stored at −80 °C until measurement of IL-10 and TNF-α using ELISA.

### 2.5. PBMC Isolation

On the same day as the whole heparin blood samples were drawn from the patients, PBMCs were isolated. First, the whole blood was diluted 1:1 with Phosphate-Buffered Saline (PBS) (Lonza, Basel, Switserland) supplemented with 2% heat-inactivated Fetal Calf Serum (FCS) (Biowest, Ennigerloh, Germany) at room temperature. Second, the diluted blood was carefully dripped on the porous membrane of the leucosep tubes (Greiner Bio-One, Kremsmünster, Austria), followed by 13 min centrifugation at 1000× *g* at 20 °C using an Eppendorf centrifuge 5810 R with the acceleration and deceleration set at 4. Third, the enriched cell fraction of PBMCs was washed twice using PBS+2%FCS. PBMCs were resuspended in RPMI-1640+20%FCS and diluted with an equal volume of ice-cold RPMI-1640+20%FCS+20%DMSO (Sigma-Aldrich) in a cryovial (maximum of 3 × 107 PBMCs/mL per cryovial). Cryovials (Corning, New York, NY, USA) were frozen using a CoolCell^®^ LX (Corning) and stored at −80 °C until used in the PBMC stimulation experiments.

### 2.6. PBMC Stimulation

PBMCs were quickly thawed and washed with culture medium consisting of RPMI-1640 supplemented with 2.5% FCS and 1% Penicillin/Streptomycin (stock 10,000 U/mL and 10,000 μg/mL respectively) (Gibco, Invitrogen, Carlsbad, CA, USA). After washing, cells were counted and diluted to a final concentration of 1 × 106 cells/mL and transferred to a 12-well suspension plate (Greiner). Visit 1 and visit 2 of the same patient were kept in the same well plate, and for each patient, two plates were prepared. Cells were left to rest for 1 h in an incubator at 37 °C and 5% CO_2_ before stimulations. Cells were stimulated with 1 μg/mL LPS or αCD3 (150 ng/mL) combined with αCD28 (100 ng/mL) (BD Biosciences, San Jose, CA, USA). After 19 h, one plate of each patient was spun down at 1200 rpm for 5 min at 20 °C in an Eppendorf centrifuge 5810 R and the supernatant was carefully discarded. Cells were restimulated with 5 ng/mL phorbol myristate acetate (PMA) (Sigma-Aldrich) and 750 ng/mL ionomycin (Sigma-Aldrich) in the presence of 1 μg/mL golgiplug (BD Biosciences) in culture medium, while plain culture medium was added to the control cells for an additional 5 h at 37 °C and 5% CO_2_. At 24 h, the experiments were ended and both plates were spun down at 1200 rpm for 5 min at 20 °C. The supernatant of the plates without restimulation was stored at −80 °C until measurement of IL-6, IL-10, IL-17a, TNF-α, IFN-γ and IL-9 using ELISA. Cells were resuspended in cold PBS and transferred to a U-bottom 96-well plate (Corning, Falcon) for FACS staining and analysis (Figure 1C,D). Left-over cells were pooled and used for fluorochrome minus one (FMO) controls, which included matched isotype controls to control for a specific signal; controls were set at the maximum of 1%.

### 2.7. FACS Analysis

Four FACS panels were used. In panel 1, PBMCs were stained with CD4 PerCP-Cyanine5.5, CD25 Alexa Fluor 488, CD127 PE-Cyanine7, FOXP3 eFluor 660 and RORγ (t) PE (All Thermo Fisher Scientific, Waltham, MA, USA). In panel 2, PBMCs were stained with CD4 PerCP-Cyanine5.5, CD69 PE and CD196 (CCR6) APC (All Thermo Fisher Scientific) and CD183 (CXCR3) Alexa Fluor 488 (BD Biosciences). In panel 3, PBMCs were stained with CD14 APC (Thermo Fisher Scientific), TLR4 PE (BD Biosciences), IL-10 Brilliant Violet 421 and TNF-α Brilliant Violet 510 (both BioLegend, San Diego, CA, USA). In panel 4, PBMCs were stained with CD4 PerCP-Cyanine5.5 (Thermo Fisher Scientific), IL-17a Alexa Fluor 488, IL-9 PE (both BD Biosciences), IFN-γ Alexa Fluor 647 (all three BioLegend), IL-10 Brilliant Violet 421 and TNF-α Brilliant Violet 510.

PBMCs in the U-bottom 96-well plate were first washed with PBS and incubated for 30 min at 4 °C with Fixable Viability dye eFluorTM 780 (Thermo Fisher Scientific) in PBS. This was followed by blocking the cells with Fc block (BD Biosciences) for 10 min at 4 °C. After blocking, the cells were incubated for 45 min with an appropriate extracellular antibody solution at 4 °C protected from light and washed with 1% bovine serum albumin (Roche) in PBS. Panel 1 was fixed overnight at 4 °C protected from light with FOXP3 fixation/permeabilization buffer (Thermo Fisher Scientific). Panel 2 was fixed with 1:4 diluted intracellular fixation buffer (Life Technologies, Themo Fisher Scientific) in PBS. Panels 3 and 4 were fixed with undiluted intracellular fixation buffer. The next day, the PBMCs were washed and blocked for 10 min at 4 °C protected from light. After blocking, an appropriate intracellular antibody solution was added for 45 min at 4 °C protected from light and washed. All panels were measured with a BD FACS Canto II flow cytometer (Becton Dickinson, Franklin Lakes, NJ, USA) and the data were analyzed using Flowlogic software Version 8 (Inivai Technologies, Mentone, Australia).

In addition, compensation beads (UltraComp eBeadsTM Plus, Life technologies) were stained with 1 μL of each antibody for 45 min at 4 °C and washed twice with FACS buffer. After washing, beads were measured with the flow cytometer to be used for compensation in the analysis. The V1 and V2 samples of each individual patient were analyzed together in the same FACS run. See Supplemental Table S1 for the titrated dilutions of the antibodies used.

### 2.8. ELISA of Whole Blood Plasma and PBMC Supernatant

TNF-α, IL-10, IL-6, IL-9, IL-17a and IFN-γ ELISA (Thermo Fisher Scientific) was performed according to the manufacturers instructions. In short, high-binding 96-well plates (Corning Costar 9018) were coated with capture antibody and incubated overnight at 4 °C. The next day, the plates were washed and blocked and the samples and standard were incubated for 2 h. After washing, a detection antibody was added to the wells and incubated for 1 h, followed by (strept)avidin-HRP for 30 min protected from light. After another round of washing, TMB solution was added and the color reaction was stopped with 2N H_2_SO_4_. Optical density was measured using a Glomax^®^ Discover Microplate Reader (Promega Corporation, Madison, WI, USA). The V1 and V2 samples of each individual patient were analyzed together on the same ELISA plate.

### 2.9. Statistical Analysis

Differences between visit 1 of the placebo group and visit 1 of the butyrate-treated group (unpaired), and between visit 2 of the placebo group and visit 2 of the butyrate-treated group, were assessed using an ordinary one-way ANOVA, with selected pairs (placebo versus butyrate visit 1; placebo versus butyrate visit 2) using Bonferroni’s post hoc test for normally distributed data or the non-parametric Kruskal–Wallis and Dunn’s post hoc test with selected pairs when data were not normally distributed. Differences within the treatment groups (paired), thus between visit 1 and visit 2 for the placebo or butyrate-treated group, were assessed using a paired Student’s *t*-test if the data were normally distributed. If not normally distributed, a Wilcoxon matched-pairs signed-rank test was used.

In addition, for each parameter, the Δvisit 2–visit 1 was calculated. Differences between Δvisit 2–visit 1 of the placebo group and Δvisit 2–visit 1 of the butyrate-treated group were assessed using an unpaired Student’s *t*-test for normally distributed data or the Mann–Whitney test for not-normally distributed data.

Results are presented as means ± SEM. Statistical analysis was performed using GraphPad Prism 8.4.3 (GraphPad Software, San Diego, CA, USA). Results were considered statistically significant when *p* < 0.05. Significant differences are shown in the figures as * *p* < 0.05, ** *p* < 0.01, *** *p* < 0.001 or **** *p* < 0.0001.

## 3. Results

### 3.1. Baseline Characteristics

Thirty-three patients (27 females and 6 males, age 50 to 74 years) participated in this study, of which sixteen received sustained-release butyrate tablets and seventeen received placebo tablets. Patient characteristics at baseline are described in Table 1. In the sustained-release butyrate-treated group, half of the patients (8 out of 16) had hand OA for less than five years, whereas this was the case for a quarter (4 out of 17) of the group given the placebo.

### 3.2. Effect of Butyrate Supplementation on Systemic Inflammation and LPS Influx

**Figure 2:**
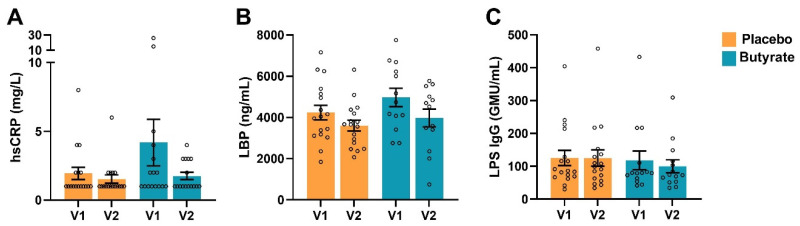
(**A**) hsCRP levels in the plasma of patients, (**B**) LBP levels and (**C**) LPS IgG levels in the serum of patients at baseline (V1) and the end of the study (V2). Orange bars indicate serum levels from patients who received the placebo and blue bars indicate patients who received butyrate. Data are presented as mean ± SEM (*n* = 17 placebo group, *n* = 13–16 butyrate group).

It was hypothesized that butyrate treatment could improve the intestinal barrier, which potentially might lead to a decreased influx of LPSs (as indicated by serum LPS IgG and LBP levels) and, as a consequence, reduced systemic inflammation (biomarker hsCRP). To test this hypothesis, systemic levels of hsCRP, LBP and LPS IgG were measured at visit 1 and visit 2. No significant changes in hsCRP were observed during the study period in either of the treatment groups (Figure 2A). Similar results were obtained for LBP and LPS-IgG, measures for LPS leakage (Figure 2B). TNF receptor 1 (TNFR1), TNF receptor 2 (TNFR2), nitrite and total nitric oxide (NO) are biomarkers shown to be elevated in association with chronic systemic inflammation. These levels were not affected by treatment with butyrate nor placebo tablets (Supplemental Figure S1), whereas nitrate also remained unaffected and IL-6 and IL-1β were below the detection limit.

### 3.3. Effect of Butyrate Supplementation on LPS-Induced Activation of Whole Blood and PBMCs

The effect of four weeks of oral sustained-release butyrate supplementation on ex vivo LPS stimulation of whole blood and PBMCs was evaluated. To monitor basal effects on monocytes, intracellular IL-10 and TNF-α measurements were performed in the CD14+ monocytes within the ionomycin–PMA-restimulated PBMC. 

LPS stimulation of the whole blood induced the release of IL-10 and TNF-α compared to the negative control (Supplemental Table S2). IL-10 and TNF-α release were both not affected by butyrate treatment for four weeks, as was the case for the placebo (Supplemental Figure S2). To study intracellular IL-10 and TNF-α in monocytes, PBMCs were restimulated with ionomycin and PMA in the presence of golgiplug. None of the used stimulations and restimulations affected cell viability (Supplemental Figure S3). LPS stimulation impacted the monocyte gating and was therefore not used; instead, medium-exposed PBMC were used to enable monocyte gating. Butyrate treatment did not affect intracellular IL-10 and TNF-α expression in the ionomycin–PMA-restimulated CD14+ monocytes, and neither did the placebo (Supplemental Figure S4A,B,E). In addition, the percentages of TLR4+ monocytes and the mean fluorescence intensity of TLR4, which indicated the level of TLR4 expression on monocytes, were not affected by the butyrate treatment nor the placebo (Supplemental Figure S4C–E).

Sustained-release butyrate treatment additionally did not affect ex vivo IL-10, TNF-α, IL-6 or IFN-γ release of LPS-stimulated PBMCs, and neither did the placebo (Supplemental Figure S5).

### 3.4. Effect of Butyrate Supplementation on Th Cells

**Figure 3:**
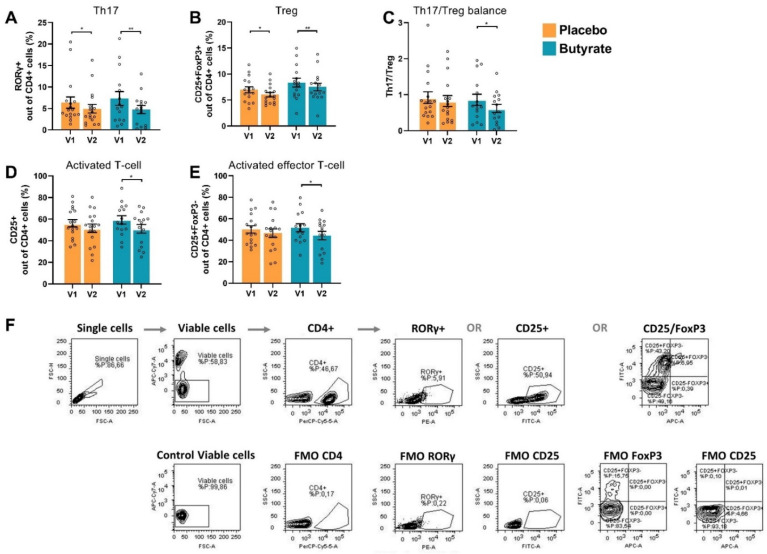
FACS analysis of αCD3/CD28-stimulated PBMCs. (**A**) Percentage of RORγ+ cells out of CD4+ cells (Th17 cells), (**B**) percentage of CD25+FoxP3+ cells out of CD4+ cells (Treg cells), (**C**) Th17/Treg balance, (**D**) percentage of CD25+ cells out of CD4+ cells (activated T-cells), (**E**) percentage of CD25+FoxP3- cells out of CD4+ cells (activated T-cells) and (**F**) the used gating strategy with corresponding fluorescence minus one (FMO) controls for a representative sample. Orange bars indicate whole blood samples from patients who received placebo and blue bars indicate patients who received butyrate. Data are presented as mean ± SEM (*n* = 17 placebo group, *n* = 15 butyrate group). Significant differences are shown as * *p* < 0.05, ** *p* < 0.01. V1: visit 1 (baseline), V2: visit 2 (end of study).

To study the effect of sustained-release butyrate supplementation on ex vivo T-cell activation, PBMCs were activated with αCD3/CD28. Within the CD4+ Th cell subset, only αCD3/CD28 stimulation of PBMCs induced a percentage of RORγ+ Th17 cells, CD25+FoxP3+ Treg, and CD25+ and CD25+FoxP3—activated effector Th cells compared to non-stimulated PBMCs (Supplemental Table S3). LPS stimulation of PBMCs did not induce these T-cell phenotypes (Supplemental Table S3). The frequencies of RORγ+ Th17 cells and CD25+FoxP3+ Treg were reduced at visit 2 compared to visit 1 after sustained-release butyrate treatment and placebo use, whereas the frequencies of CD25+ and CD25+FoxP3- activated Th cells were only reduced after the butyrate treatment and not after the placebo (Figure 3). However, no significant difference was observed between the butyrate group and the placebo group when comparing the ΔV2–V1 of both groups (Supplemental Table S3). In addition, the Th17/Treg balance was only reduced in the patient group treated with sustained-release butyrate, which was not observed after placebo use, although again no significant difference was observed when comparing the ΔV2–V1 of the butyrate- and the placebo-treated groups (Supplemental Table S3).

**Figure 4:**
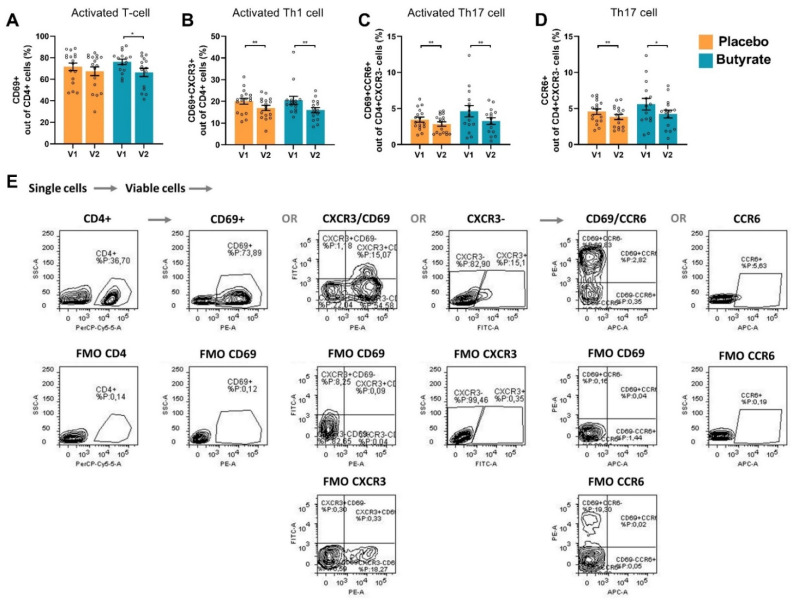
FACS analysis of αCD3/CD28-stimulated PBMCs. (**A**) Percentage of CD69+ cells out of CD4+ cells (Activated T-cells), (**B**) percentage of CD69+CXCR3+ cells out of CD4+ cells (activated Th1 cells), (**C**) percentage of CD69+CCR6+ cells out of CD4+CXCR3- cells (activated Th17 cells), (**D**) percentage of CCR6+ cells out of CD4+CXCR3- cells (Th17 cells) and (**E**) the used gating strategy with corresponding fluorescence minus one (FMO) controls for a representative sample. Orange bars indicate whole blood samples from patients who received placebo and blue bars indicate patients who received butyrate. Data are presented as mean ± SEM (*n* = 17 placebo group, *n* = 15 butyrate group). Significant differences are shown as * *p* < 0.05, ** *p* < 0.01. V1: visit 1 (baseline), V2: visit 2 (end of study).

Furthermore, αCD3/CD28 stimulation of PBMCs enhanced the frequency of CD69+-activated Th cells, CCR6+CXCR3- Th17 cells, CD69+CCR6+CXCR3-activated Th17 cells and CD69+CXCR3+ Th1 cells within the CD4+ population compared to non-stimulated PBMCs (Supplemental Table S3). LPS stimulation of PBMCs did not induce these T-cell phenotypes (Supplemental Table S3). The percentage of CD69+-activated Th cells was reduced after use of sustained-release butyrate tablets and not after use of the placebo (Figure 4A,E). However, no significant effect was observed when comparing the ΔV2–V1 of CD69+-activated Th cells between the placebo and the butyrate group (Supplemental Table S2). Similar to the percentage of intracellular RORγ+-expressing Th cells, the frequency of Th17 cells was also phenotyped via their surface marker expression. The percentages of CCR6+CXCR3- Th17 cells and CD69+CCR6+CXCR3-activated Th17 cells were reduced at visit 2 compared to visit 1 in the ex vivo αCD3/CD28-activated PBMCs of OA patients receiving sustained-release butyrate supplementation and after placebo use as well (Figure 4C,D). In addition, the frequency of CD69+CXCR3+-activated Th1 cells was reduced both after use of sustained-release butyrate as well as the placebo (Figure 4B).

**Figure 5:**
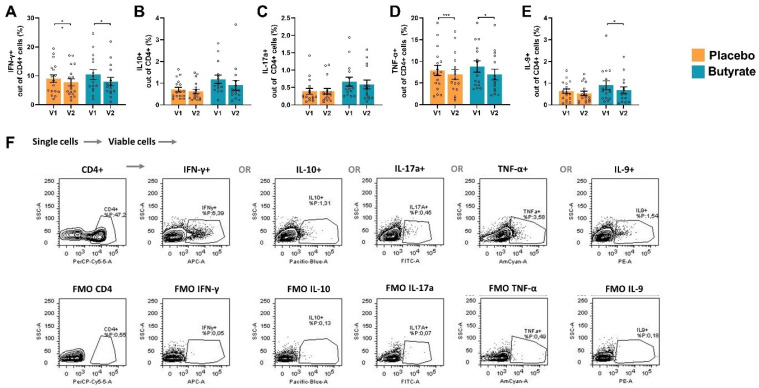
FACS analysis of αCD3/CD28-stimulated PBMCs which were restimulated with PMA, ionomycin and golgiplug. (**A**) Percentage of IFN-γ+ cells out of CD4+ cells, (**B**) percentage of IL-10+ cells out of CD4+ cells, (**C**) percentage of IL-17a+ cells out of CD4+ cells, (**D**) percentage of TNF-α+ cells out of CD4+ cells, (**E**) percentage of IL-9+ cells out of CD4+ cells and (**F**) the used gating strategy with corresponding fluorescence minus one (FMO) controls for a representative sample. Orange bars indicate whole blood samples from patients who received placebo and blue bars indicate patients who received butyrate. Data are presented as mean ± SEM (*n* = 17 placebo group, *n* = 15 butyrate group). Significant differences are shown as * *p* < 0.05, *** *p* < 0.001. V1: visit 1 (baseline), V2: visit 2 (end of study).

Even though some T-cell phenotypes were affected by the treatments, no effects of the sustained-release butyrate nor placebo were observed on the release of regulatory cytokine IL-10 and inflammatory cytokines TNF-α, IL-6, IFN-γ, IL-9 or IL-17a by αCD3/CD28-stimulated PBMCs (Supplemental Table S3). However, when studying intracellular cytokine expression in CD4+ T-cells within the αCD3/CD28-stimulated PBMCs, significant effects were observed (Figure 5). These PBMCs were restimulated with ionomycin and PMA in the presence of golgiplug, enabling the intracellular measurement of IL-10+, TNF-α+, IL-6+, IFN-γ+, IL-9+ or IL-17a+ in Th cells compared to non-activated PBMCs (Supplemental Table S3). Neither the sustained-release butyrate nor the placebo affected the percentage of IL-10+ or IL-17a+ cells, but both reduced the percentage of IFN-γ+ and TNF-α+ Th cells. In addition, sustained-release butyrate, but not the placebo, reduced the percentage of IL-9+ Th cells. However, no significant effect was observed comparing the ΔV2–V1 of the percentage IL-9+ Th cells between the placebo and the butyrate group (Supplemental Table S2).

## 4. Discussion

The purpose of this study was to investigate the effects of four to five weeks of sustained-release butyrate treatment on systemic inflammation and immune function, as studied using ex vivo stimulation of the whole blood and PBMCs of hand OA patients.

The pathophysiology of hand OA involves multiple factors. Increasing evidence suggests a compromised intestinal barrier and low-grade systemic inflammation as contributing factors [2,7]. In the current study, hand OA patients were provided with sustained-release butyrate tablets and it was aimed to deliver a butyrate concentration in the intestinal lumen high enough for a pharmacological effect leading to reduced systemic inflammation via improvement of, among other factors, the intestinal barrier function. However, in the present study, butyrate did not affect systemic inflammation marker hsCRP. Neither serum LBP or IgG LPS concentrations, which both are indirect measures for intestinal LPS leakage, were affected. LBP is a protein which is synthesized by the liver in response to inflammatory stimuli, particularly LPSs, whereas IgG LPS is an antibody which is produced in response to LPSs. The effect of butyrate treatment on hsCRP, LBP or IgG LPS has not been studied before in clinical trials. Among markers associated with low-grade systemic inflammation such as IL-6, IL-1β and hsCRP, in this study only hsCRP was detectable, although in low concentrations. Systemic low-grade inflammation, as determined by hsCRP levels, becomes more pronounced in patients having more severe OA [10]. It can be hypothesized that for this reason it was not possible to detect an effect of the intervention on low-grade inflammation in this patient category. In addition, even though hsCRP levels can be elevated in OA patients, the increase compared to healthy controls is relatively small [10]. The same accounts for LBP [47,48,49]. Beyond these parameters, other OA-associated systemic inflammation markers like NO and TNFR also remained unaffected. Therefore, the main objective to use a dose of butyrate high enough to suppress LPS leakage and thus systemic inflammation may have failed in the current study group with patients receiving 600 mg butyrate per day. Other clinical trials have used dosages up to 4 g butyrate per day, indicating that it would be safe to increase the dosage of sustained-release butyrate tablets in future studies, although it should be noted that the formulations used in the other trials were completely different, namely being colon-targeted or immediate release. We developed a sustained-release butyrate tablet which slowly releases steady amounts of butyrate along the whole small intestine to reach a local pharmacologically active concentration. Nonetheless, it remains to be revealed if indeed a pharmacologically active concentration was present and whether the exposure time was sufficient to achieve a pharmacological effect on systemic inflammation markers. The absence of an effect on systemic inflammation markers and LPS leakage markers might suggest that the designed formulation was ineffective, possibly due to one of the aforementioned reasons, although it might also be that the four-week treatment duration was too short to achieve these effects. 

The systemic immunomodulatory effect of butyrate treatment was also studied in ex vivo stimulated whole blood or PBMCs. These stimulations were performed to give more insight in the potential of butyrate as a treatment for patients with OA, because these stimulations show the inflammatory potential of different immune cells, such as monocytes and T-cells. Monocytes are key effector cells of the immune system and precursors of macrophages such as those present in the inflamed synovia of OA patients. Furthermore, emerging evidence shows that infiltration of monocytes into the synovial tissues of knee OA patients is part of the pathogenesis [50], as well as elevated monocyte activation systemically [51]. In different in vitro studies, it was observed that butyrate inhibits the release of pro-inflammatory cytokines and induced the release of regulatory cytokines by monocytes, which could be beneficial in OA patients [37,52]. Similar to macrophages, monocytes are very sensitive to LPS activation. However, in the present study we did not observe any effect of the butyrate treatment on ex vivo LPS stimulation of whole blood cells nor PBMCs, indicating that the sustained-release tablets did not affect monocytes and LPS-induced cytokine release. Contrary, another study showed that in vivo butyrate treatment did decrease ex vivo oxLDL or β-glucan-induced trained immunity in the monocytes of obese males [53]. Even though this study did not concern OA patients and the monocytes were first trained before LPS or PAM3CSK4 activation, the main difference may be the dose of butyrate given, since in the other study a twice-daily intake of 4 g butyrate for four weeks was studied, which is approximately 13 times higher than the dosage used in the present study. Further, the release characteristics of butyrate from the formulations were different between both studies as well. From animal studies and in vitro studies, it is known that short-chain fatty acids can affect the balance of pro-inflammatory M1- and anti-inflammatory M2-type macrophages in favor of M2, which might ameliorate inflammatory effects [54]. Additionally, eight weeks of treatment with 100 mM butyrate enemas decreased nuclear translocation of NK-kappaB in the macrophages of patients with ulcerative colitis [55]. We hypothesize that a higher dosage of sustained-release butyrate might have anti-inflammatory effects on monocytes and macrophages, which needs to be confirmed in future studies. 

T-cells play a central role in the adaptive immune system. Similar to monocytes, T-cell infiltration into the synovial tissues of OA patients contributes to the disease’s pathogenesis, as well as T-cell subset imbalances and altered production of cytokines by T-cells systemically. For example, it was observed that the percentage of Treg (IL7R-CD25+) cells is decreased in peripheral blood of patients with inflammatory knee OA [56] and the percentages of CD4+CD8-IL9+, CD4+CD8-IFNγ and CD4+CD8-IL17a+ cells and serum levels of IL-9, IFN-γ, IL-17a and IL-6 are increased [24,57]. 

In the current study, serum levels of IL-6 remained below detection and the PBMCs of this moderate hand OA patient group did not secrete any of the measured cytokines in absence of stimulation. However, upon stimulation with αCD3/CD28, we were able to study the effect of four weeks of sustained-release butyrate treatment on ex vivo T-cell activation. Butyrate treatment reduced the percentage of activated CD69+CD4+, CD25+CD4+ and activated effector CD25+FoxP3- Th cells and produced a lowered Th17/Treg ratio, shifting the balance in favor of Treg. However, no significant difference was observed when comparing the ΔV2–V1 between the butyrate and placebo groups. Therefore, studies in bigger patient groups are required to fully exclude the involvement of a placebo effect. Reduced generic T-cell activation and an improved Th17/Treg balance could be beneficial for OA patients, in whom Th1, Th17 and Th9 activation is known to contribute to disease pathology along with an increased Th17/Treg balance [23,25]. Even though the activation status of the Th cells was suppressed in the butyrate group, the cytokine release of the ex vivo activated PBMC remained unaffected. It may be that the effect on activation was too small to also suppress cytokine secretion or that beyond Th cells, other cells like natural killer cells and cytotoxic T-cells also contributed to the secretion of these cytokines [58]. However, when studying intracellular cytokine expression within the Th cells of αCD3/CD28-activated PBMCs, the percentage of IL9+ cells was also reduced by the butyrate treatment. Intracellular IFN-γ and TNF-α expression were also reduced, but this also applied for the placebo group, while the lowering of IL-9+ Th9 cells was selective for the butyrate group, although a placebo effect cannot be excluded since the ΔV2–V1 between the butyrate and placebo group did not differ. Beyond pro-inflammatory cytokines like IFN-γ, IL-17a and TNF-α, IL-9 is also an important player in OA disease progression. IL-9 is being recognized as a systemic biomarker in knee OA severity. It was shown that the number of Th9 cells in circulation was positively associated with elevated CRP levels and that the number of Th9 cells and serum IL-9 concentrations in patients with OA were positively related with a loss of daily functioning [24]. Therefore, these results imply that our sustained-release butyrate treatment does have a beneficial effect, reducing the state of inflammation in OA patients at least at the level of the T-cells.

The barrier protective and anti-inflammatory effects of butyrate are well studied, and previously we have shown in in vitro mucosal immune models that butyrate can protect against inflammatory-induced barrier disruption and silence immune cell activation within 24 h; both were linked to the HDAC inhibitory capacities of butyrate [33,42]. Future studies are warranted to analyze the mechanism of action of butyrate sustained-release tablets on T-cell function in OA patients.

In addition to the parameters mentioned above, the butyrate and placebo treatment both lowered the frequency of Treg cells and activated Th1 cells, Th17 cells, IFNγ+CD4+ cells and TNFα+CD4+ cells. The patient inclusion of the butyrate and placebo group was done on regular a basis during the year at one location, so the inclusion period and location were similar between the groups. Additionally, the general patient characteristics did not differ between groups. The excipients used in the formulation are not known to have any pharmacological effects; however, interference of the excipients with the intestinal microbiome can be possible. Indeed, nonfermentable fiber hydroxypropyl methylcellulose may have beneficially affected the microbiome [59,60,61], which may have caused immunomodulatory effects. 

The present study has several limitations which should be taken into account. The study was set up as a proof-of-concept study and the study groups may have been too small, limiting the statistical power for these secondary parameters determining immune function. In general, no placebo-controlled studies are available in this field and our study is one of the first explorative studies measuring several inflammatory markers. Data collected in the current study will help to enable power calculations of future clinical trials in this area. Furthermore, OA patients were given two 150 mg butyrate (as calcium) sustained-release tablets twice daily. This may have been too limited for full pharmacological effectiveness as doses up to 4 g were used as treatment in inflammatory bowel disease [62,63,64]. Butyrate is known to be readily absorbed via active transport in the intestine [31]. In addition, butyrate is known to be a fuel source for IEC, although part of the butyrate could also be released into the lamina propria, hence reaching underlying immune cells. As only a small percentage of the butyrate available in the lumen of the intestine will reach the portal vein and will be further metabolized in the liver, butyrate availability in systemic circulation therefore is very low [31,65]. The dose in the tablets is relatively low and only meant to act locally in the gut mucosal tissue, mainly targeting the enterocytes [46]. The sustained-release butyrate tablets used in this study should thus theoretically be able to release enough butyrate for a pharmacological response in the small intestine, but local butyrate concentrations were not measured. It could be that the butyrate dosage was too low to exert the optimal effect, that the release characteristics of the tablet or the formulation itself were not optimal and/or it could be that the exposure time of the butyrate was too low. However, since the current study shows beneficial effects in the T-cells of hand OA patients, increasing the dosage and/or extending the period of intervention may help to reduce severity and/or slow down disease progression in this patient group. This should therefore be investigated further in future studies, also including analyses of other inflammatory markers like IL-8, oxidative stress markers and a broader panel of acute phase proteins.

## 5. Conclusions

This double-blind placebo-controlled randomized clinical trial showed that the sustained-release butyrate treatment reduced the inflammatory potential of Th cells in hand OA patients, as indicated by a reduced percentage of activated Th cells and IL-9-expressing Th9 cells and an improved Th17/Treg balance within ex vivo αCD3/CD28-activated PBMCs. This could contribute to restoring the immune balance in hand OA patients, which might benefit the patient by reducing their inflammatory status.

## Figures and Tables

**Table 1:** Patient characteristics at baseline, if applicable values are given as the mean ± SD. K&L: Kellgren and Lawrence.

	Placebo (*n* = 17)	Sustained-Release Butyrate (*n* = 16)
Age (years)	63.3 ± 8.3	61.6 ± 5.0
BMI (kg/m^2^)	25.5 ± 2.7	26.0 ± 2.5
Female (*n*) Male (*n*)	13 4	14 2
Disease duration < 5 years (*n*)	4	8
Disease duration ≥ 5 years (*n*) Number of joints K&L ≥ 2 (0–30 joints)	13 8 ± 5	8 8 ± 4

## Data Availability

The raw data supporting the conclusions of this article will be made available by the authors on request.

## Supplementary Materials

The following supporting information can be downloaded at: https://www.mdpi.com/article/10.3390/nu16193384/s1, Figure S1: (A) TNF receptor 1 (TNFR1), (B) TNF receptor 2 (TNFR2), (C) Nitrite, and (D) total nitric oxide (NO) at baseline (V1) and the end of the study (V2); Figure S2: Ex vivo LPS stimulation of whole blood samples to induce IL-10; Figure S3: Viability of PBMCs after stimulation with either LPS or αCD3/CD28 with and without restimulation (PMA and Ionomycine in presence of golgiplug) and the negative control; Figure S4: FACS analysis of monocytes within restimulated PBMCs; Figure S5: IL-10 (A), TNF-α (B), IL-6 (C) and IFN-γ (D) release of LPS-stimulated PBMCs measured with ELISA; Table S1: Overview titrated dilution of FACS antibodies; Table S2: ELISA data of LPS-stimulated whole blood and negative control and FACS data of restimulated PBMCs and negative control; Table S3: FACS and ELISA data of LPS-stimulated, αCD3/CD28-stimulated, αCD3/CD28-stimulated+restimulated PBMCs and negative control.

## References

[1] Biver E., Berenbaum F., Valdes A.M., Araujo de Carvalho I., Bindels L.B., Brandi M.L., Calder P.C., Castronovo V., Cavalier E., Cherubini A. (2019). Gut Microbiota and Osteoarthritis Management: An Expert Consensus of the European Society for Clinical and Economic Aspects of Osteoporosis, Osteoarthritis and Musculoskeletal Diseases (ESCEO). Ageing Res. Rev..

[2] Favazzo L.J., Hendesi H., Villani D.A., Soniwala S., Dar Q.A., Schott E.M., Gill S.R., Zuscik M.J. (2020). The Gut Microbiome-Joint Connection: Implications in Osteoarthritis. Curr. Opin. Rheumatol..

[3] Berthelot J.M., Sellam J., Maugars Y., Berenbaum F. (2019). Cartilage-Gut-Microbiome Axis: A New Paradigm for Novel Therapeutic Opportunities in Osteoarthritis. RMD Open.

[4] Boer C.G., Radjabzadeh D., Medina-Gomez C., Garmaeva S., Schiphof D., Arp P., Koet T., Kurilshikov A., Fu J., Ikram M.A. (2019). Intestinal Microbiome Composition and Its Relation to Joint Pain and Inflammation. Nat. Commun..

[5] Huang Z., Kraus V.B. (2016). Does Lipopolysaccharide-Mediated Inflammation Have a Role in OA?. Nat. Rev. Rheumatol..

[6] Jackson M.A., Verdi S., Maxan M.E., Shin C.M., Zierer J., Bowyer R.C.E., Martin T., Williams F.M.K., Menni C., Bell J.T. (2018). Gut Microbiota Associations with Common Diseases and Prescription Medications in a Population-Based Cohort. Nat. Commun..

[7] Sun C., Zhou X., Guo T., Meng J. (2023). The Immune Role of the Intestinal Microbiome in Knee Osteoarthritis: A Review of the Possible Mechanisms and Therapies. Front. Immunol..

[8] Huang Z.Y., Stabler T., Pei F.X., Kraus V.B. (2016). Both Systemic and Local Lipopolysaccharide (LPS) Burden Are Associated with Knee OA Severity and Inflammation. Osteoarthr. Cartil..

[9] Huang Z., Perry E., Huebner J.L., Katz B., Li Y., Kraus V.B. (2018). Biomarkers of Inflammation—LBP and TLR– Predict Progression of Knee Osteoarthritis in the DOXY Clinical Trial. Osteoarthr. Cartil..

[10] Jin X., Beguerie J.R., Zhang W., Blizzard L., Otahal P., Jones G., Ding C. (2015). Circulating C Reactive Protein in Osteoarthritis: A Systematic Review and Meta-Analysis. Ann. Rheum. Dis..

[11] Kaneko S., Satoh T., Chiba J., Ju C., Inoue K., Kagawa J. (2000). Interleukin-6 and Interleukin-8 Levels in Serum and Synovial Fluid of Patients with Osteoarthritis. Cytokines Cell. Mol. Ther..

[12] Otterness I.G., Weiner E., Swindell A.C., Zimmerer R.O., Ionescu M., Poole A.R. (2001). An Analysis of 14 Molecular Markers for Monitoring Osteoarthritis. Relationship of the Markers to Clinical End-Points. Osteoarthr. Cartil..

[13] Otterness I.G., Swindell A.C., Zimmerer R.O., Poole A.R., Ionescu M., Weiner E. (2000). An Analysis of 14 Molecular Markers for Monitoring Osteoarthritis: Segregation of the Markers into Clusters and Distinguishing Osteoarthritis at Baseline. Osteoarthr. Cartil..

[14] Toncheva A., Remichkova M., Ikonomova K., Dimitrova P., Ivanovska N. (2009). Inflammatory Response in Patients with Active and Inactive Osteoarthritis. Rheumatol. Int..

[15] Pearle A.D., Scanzello C.R., George S., Mandi L.A., Dicarlo E.F., Peterson M., Sculco T.P., Crow M.K. (2007). Elevated High-Sensitivity C-Reactive Protein Levels Are Associated with Local Inflammatory Findings in Patients with Osteoarthritis. Osteoarthr. Cartil..

[16] Tilg H., Moschen A.R. (2014). Microbiota and Diabetes: An Evolving Relationship. Gut.

[17] Kriss M., Hazleton K.Z., Nusbacher N.M., Martin C.G., Lozupone C.A. (2018). Low Diversity Gut Microbiota Dysbiosis: Drivers, Functional Implications and Recovery. Curr. Opin. Microbiol..

[18] Ohigashi S., Sudo K., Kobayashi D., Takahashi O., Takahashi T., Asahara T., Nomoto K., Onodera H. (2013). Changes of the Intestinal Microbiota, Short Chain Fatty Acids, and Fecal PH in Patients with Colorectal Cancer. Dig. Dis. Sci..

[19] Haseeb A., Haqqi T.M. (2013). Immunopathogenesis of Osteoarthritis. Clin. Immunol..

[20] Lopes E.B.P., Filiberti A., Husain S.A., Humphrey M.B. (2017). Immune Contributions to Osteoarthritis. Curr. Osteoporos. Rep..

[21] de Lange-Brokaar B.J.E., Ioan-Facsinay A., van Osch G.J.V.M., Zuurmond A.M., Schoones J., Toes R.E.M., Huizinga T.W.J., Kloppenburg M. (2012). Synovial Inflammation, Immune Cells and Their Cytokines in Osteoarthritis: A Review. Osteoarthr. Cartil..

[22] Orlowsky E.W., Kraus V.B. (2015). The Role of Innate Immunity in Osteoarthritis: When Our First Line of Defense Goes on the Offensive. J. Rheumatol..

[23] Li Y.S., Luo W., Zhu S.A., Lei G.H. (2017). T Cells in Osteoarthritis: Alterations and Beyond. Front. Immunol..

[24] Qi C., Shan Y., Wang J., Ding F., Zhao D., Yang T., Jiang Y. (2016). Circulating T Helper 9 Cells and Increased Serum Interleukin-9 Levels in Patients with Knee Osteoarthritis. Clin. Exp. Pharmacol. Physiol..

[25] Ye X., Lu Q., Yang A., Rao J., Xie W., He C., Wang W., Li H., Zhang Z. (2021). *MiR*-206 Regulates the Th17/Treg Ratio during Osteoarthritis. Mol. Med..

[26] Askari A., Naghizadeh M.M., Homayounfar R., Shahi A., Afsarian M.H., Paknahad A., Kennedy D., Ataollahi M.R. (2016). Increased Serum Levels of IL-17A and IL-23 Are Associated with Decreased Vitamin D3 and Increased Pain in Osteoarthritis. PLoS ONE.

[27] Li S., Wan J., Anderson W., Sun H., Zhang H., Peng X., Yu Z., Wang T., Yan X., Smith W. (2016). Downregulation of IL-10 Secretion by Treg Cells in Osteoarthritis Is Associated with a Reduction in Tim-3 Expression. Biomed. Pharmacother..

[28] Ghouri A., Conaghan P.G. (2019). Update on Novel Pharmacological Therapies for Osteoarthritis. Ther. Adv. Musculoskelet. Dis..

[29] Osani M.C., Vaysbrot E.E., Zhou M., McAlindon T.E., Bannuru R.R. (2019). Duration of Symptom Relief and Early Trajectory of Adverse Events for Oral Nonsteroidal Antiinflammatory Drugs in Knee Osteoarthritis: A Systematic Review and Meta-analysis. Arthritis Care Res..

[30] Canani R.B., Costanzo M.D., Leone L., Pedata M., Meli R., Calignano A. (2011). Potential Beneficial Effects of Butyrate in Intestinal and Extraintestinal Diseases. World J. Gastroenterol..

[31] Tan J., McKenzie C., Potamitis M., Thorburn A.N., Mackay C.R., Macia L. (2014). The Role of Short-Chain Fatty Acids in Health and Disease.

[32] Guilloteau P., Martin L., Eeckhaut V., Ducatelle R., Zabielski R., Van Immerseel F. (2010). From the Gut to the Peripheral Tissues: The Multiple Effects of Butyrate. Nutr. Res. Rev..

[33] Korsten S.G.P.J., Vromans H., Garssen J., Willemsen L.E.M. (2023). Butyrate Protects Barrier Integrity and Suppresses Immune Activation in a Caco-2/PBMC Co-Culture Model While HDAC Inhibition Mimics Butyrate in Restoring Cytokine-Induced Barrier Disruption. Nutrients.

[34] Usami M., Kishimoto K., Ohata A., Miyoshi M., Aoyama M., Fueda Y., Kotani J. (2008). Butyrate and Trichostatin A Attenuate Nuclear Factor ΚB Activation and Tumor Necrosis Factor α Secretion and Increase Prostaglandin E2 Secretion in Human Peripheral Blood Mononuclear Cells. Nutr. Res..

[35] Asarat M., Apostolopoulos V., Vasiljevic T., Donkor O. (2016). Short-Chain Fatty Acids Regulate Cytokines and Th17/Treg Cells in Human Peripheral Blood Mononuclear Cells In Vitro. Immunol. Investig..

[36] Asarat M., Vasiljevic T., Apostolopoulos V., Donkor O. (2015). Short-Chain Fatty Acids Regulate Secretion of IL-8 from Human Intestinal Epithelial Cell Lines in Vitro. Immunol. Investig..

[37] Säemann M.D., Böhmig G.A., Österreicher C.H., Burtscher H., Parolini O., Diakos C., Stöckl J., Hörl W.H., Zlabinger G.J. (2000). Anti-inflammatory Effects of Sodium Butyrate on Human Monocytes: Potent Inhibition of IL-12 and Up-regulation of IL-10 Production. FASEB J..

[38] Cox M.A., Jackson J., Stanton M., Rojas-Triana A., Bober L., Laverty M., Yang X., Zhu F., Liu J., Wang S. (2009). Short-Chain Fatty Acids Act as Antiinflammatory Mediators by Regulating Prostaglandin E2 and Cytokines. World J. Gastroenterol..

[39] Segain J.P., Raingeard de la Blétière D., Bourreille A., Leray V., Gervois N., Rosales C., Ferrier L., Bonnet C., Blottière H.M., Galmiche J.P. (2000). Butyrate Inhibits Inflammatory Responses through NFkappaB Inhibition: Implications for Crohn’s Disease. Gut.

[40] D’Souza W.N., Douangpanya J., Mu S., Jaeckel P., Zhang M., Maxwell J.R., Rottman J.B., Labitzke K., Willee A., Beckmann H. (2017). Differing Roles for Short Chain Fatty Acids and GPR43 Agonism in the Regulation of Intestinal Barrier Function and Immune Responses. PLoS ONE.

[41] Fukae J., Amasaki Y., Yamashita Y., Bohgaki T., Yasuda S., Jodo S., Atsumi T., Koike T. (2005). Butyrate Suppresses Tumor Necrosis Factor α Production by Regulating Specific Messenger RNA Degradation Mediated through a Cis-Acting AU-Rich Element. Arthritis Rheum..

[42] Korsten S.G.P.J., Peracic L., van Groeningen L.M.B., Diks M.A.P., Vromans H., Garssen J., Willemsen L.E.M. (2022). Butyrate Prevents Induction of CXCL10 and Non-Canonical IRF9 Expression by Activated Human Intestinal Epithelial Cells via HDAC Inhibition. Int. J. Mol. Sci..

[43] Böcker U., Nebe T., Herweck F., Holt L., Panja A., Jobin C., Rossol S., Sartor R.B., Singer M.V. (2003). Butyrate Modulates Intestinal Epithelial Cell-Mediated Neutrophil Migration. Clin. Exp. Immunol..

[44] Kil Lee S., Kim T.I., Kim Y.K., Choi C.H., Yang K.M., Chae B., Kim W.H. (2005). Cellular Differentiation-Induced Attenuation of LPS Response in HT-29 Cells Is Related to the down-Regulation of TLR4 Expression. Biochem. Biophys. Res. Commun..

[45] Ohata A., Usami M., Miyoshi M. (2005). Short-Chain Fatty Acids Alter Tight Junction Permeability in Intestinal Monolayer Cells via Lipoxygenase Activation. Nutrition.

[46] Korsten S.G.P.J., Smits E.A.W., Garssen J., Vromans H. (2019). Modeling of the Luminal Butyrate Concentration to Design an Oral Formulation Capable of Achieving a Pharmaceutical Response. PharmaNutrition.

[47] Hasegawa S., Goto S., Tsuji H., Okuno T., Asahara T., Nomoto K., Shibata A., Fujisawa Y., Minato T., Okamoto A. (2015). Intestinal Dysbiosis and Lowered Serum Lipopolysaccharide-Binding Protein in Parkinson’s Disease. PLoS ONE.

[48] Rojo Ó.P., San Román A.L., Arbizu E.A., Martínez A.D.L.H., Sevillano E.R., Martínez A.A. (2007). Serum Lipopolysaccharide-Binding Protein in Endotoxemic Patients with Inflammatory Bowel Disease. Inflamm. Bowel Dis..

[49] Myc A., Buck J., Gonin J., Reynolds B., Hammerling U., Emanuel D. (1997). The Level of Lipopolysaccharide-Binding Protein Is Significantly Increased in Plasma in Patients with the Systemic Inflammatory Response Syndrome. Clin. Diagn. Lab. Immunol..

[50] Gómez-Aristizábal A., Gandhi R., Mahomed N.N., Marshall K.W., Viswanathan S. (2019). Synovial Fluid Monocyte/Macrophage Subsets and Their Correlation to Patient-Reported Outcomes in Osteoarthritic Patients: A Cohort Study. Arthritis Res. Ther..

[51] Loukov D., Karampatos S., Maly M.R., Bowdish D.M.E. (2018). Monocyte Activation Is Elevated in Women with Knee-Osteoarthritis and Associated with Inflammation, BMI and Pain. Osteoarthr. Cartil..

[52] Larasati R.A., Harbuwono D.S., Rahajeng E., Pradipta S., Nuraeni H.S., Susilowati A., Wibowo H. (2019). The Role of Butyrate on Monocyte Migration and Inflammation Response in Patient with Type 2 Diabetes Mellitus. Biomedicines.

[53] Cleophas M.C.P., Ratter J.M., Bekkering S., Quintin J., Schraa K., Stroes E.S., Netea M.G., Joosten L.A.B. (2019). Effects of Oral Butyrate Supplementation on Inflammatory Potential of Circulating Peripheral Blood Mononuclear Cells in Healthy and Obese Males. Sci. Rep..

[54] Wang Z., Zhang X., Zhu L., Yang X., He F., Wang T., Bao T., Lu H., Wang H., Yang S. (2020). Inulin Alleviates Inflammation of Alcoholic Liver Disease via SCFAs-Inducing Suppression of M1 and Facilitation of M2 Macrophages in Mice. Int. Immunopharmacol..

[55] Lührs H., Gerke T., Müller J.G., Melcher R., Schauber J., Boxberger F., Scheppach W., Menzel T. (2002). Butyrate Inhibits NF-ΚB Activation in Lamina Propria Macrophages of Patients with Ulcerative Colitis. Scand. J. Gastroenterol..

[56] Penatti A., Facciotti F., De Matteis R., Larghi P., Paroni M., Murgo A., De Lucia O., Pagani M., Pierannunzii L., Truzzi M. (2017). Differences in Serum and Synovial CD4+ T Cells and Cytokine Profiles to Stratify Patients with Inflammatory Osteoarthritis and Rheumatoid Arthritis. Arthritis Res. Ther..

[57] Imamura M., Ezquerro F., Marcon Alfieri F., Vilas Boas L., Tozetto-Mendoza T.R., Chen J., Özçakar L., Arendt-Nielsen L., Rizzo Battistella L. (2015). Serum Levels of Proinflammatory Cytokines in Painful Knee Osteoarthritis and Sensitization. Int. J. Inflam..

[58] Lawlor N., Nehar-Belaid D., Grassmann J.D.S., Stoeckius M., Smibert P., Stitzel M.L., Pascual V., Banchereau J., Williams A., Ucar D. (2021). Single Cell Analysis of Blood Mononuclear Cells Stimulated Through Either LPS or Anti-CD3 and Anti-CD28. Front. Immunol..

[59] Tamargo A., Cueva C., Alvarez M.D., Herranz B., Moreno-Arribas M.V., Laguna L. (2019). Physical Effects of Dietary Fibre on Simulated Luminal Flow, Studied by In Vitro Dynamic Gastrointestinal Digestion and Fermentation. Food Funct..

[60] Cox L.M., Cho I., Young S.A., Anderson W.H.K., Waters B.J., Hung S.C., Gao Z., Mahana D., Bihan M., Alekseyenko A.V. (2013). The Nonfermentable Dietary Fiber Hydroxypropyl Methylcellulose Modulates Intestinal Microbiota. FASEB J..

[61] Naimi S., Viennois E., Gewirtz A.T., Chassaing B. (2021). Direct Impact of Commonly Used Dietary Emulsifiers on Human Gut Microbiota. Microbiome.

[62] Scheppach W., Sommer H., Kirchner T., Paganelli G.M., Bartram P., Christl S., Richter F., Dusel G., Kasper H. (1992). Effect of Butyrate Enemas on the Colonic Mucosa in Distal Ulcerative Colitis. Gastroenterology.

[63] Hamer H.M., Jonkers D.M.A.E., Vanhoutvin S.A.L.W., Troost F.J., Rijkers G., de Bruïne A., Bast A., Venema K., Brummer R.J.M. (2010). Effect of Butyrate Enemas on Inflammation and Antioxidant Status in the Colonic Mucosa of Patients with Ulcerative Colitis in Remission. Clin. Nutr..

[64] Di Sabatino A., Morera R., Ciccocioppo R., Cazzola P., Gotti S., Tinozzi F.P., Tinozzi S., Corazza G.R. (2005). Oral Butyrate for Mildly to Moderately Active Crohn’s Disease. Aliment. Pharmacol. Ther..

[65] Liu H., Wang J., He T., Becker S., Zhang G., Li D., Ma X. (2018). Butyrate: A Double-Edged Sword for Health?. Adv. Nutr..

