# Supplementary material for: A Sustained-Release Butyrate Tablet Suppresses Ex Vivo T Helper Cell Activation of Osteoarthritis Patients in a Double-Blind Placebo-Controlled Randomized Trial
Source: Nutrients. 2024 Oct 4;16(19):3384. doi: 10.3390/nu16193384 (PMC11478393; doi:10.3390/nu16193384)
Supplement: Supplementary file 1 [file nutrients-16-03384-s001.zip › nutrients-3231751-supplementary.pdf]

Supplemental Table S1. Overview titrated dilution of FACS antibodies.

Antibody	Brand	Dilution
CD4 PerCP-Cyanine5.5	Thermo Fisher Scientific	1:80
CD25 Alexa Fluor 488	Thermo Fisher Scientific	1:40
CD127 PE-Cyanine7	Thermo Fisher Scientific	1:80
FOXP3 eFluor 660	Thermo Fisher Scientific	1:320
RORγ (t) PE	Thermo Fisher Scientific	1:640
CD69 PE	Thermo Fisher Scientific	1:80
CD196 (CCR6) APC	Thermo Fisher Scientific	1:160
CD183 (CXCR3) Alexa Fluor 488	BD Biosciences	1:40
CD14 APC	Thermo Fisher Scientific	1:160
TLR4 PE	BD Biosciences	1:80
IL-10 Brilliant Violet 421	Biolegend	1:160
TNF-α Brilliant Violet 510	Biolegend	1:50
IL-17A Alexa Fluor 488	BD Biosciences	1:40
IL-9 PE	BD Biosciences	1:320
IFN-γ Alexa Fluor 647	Biolegend	1:120

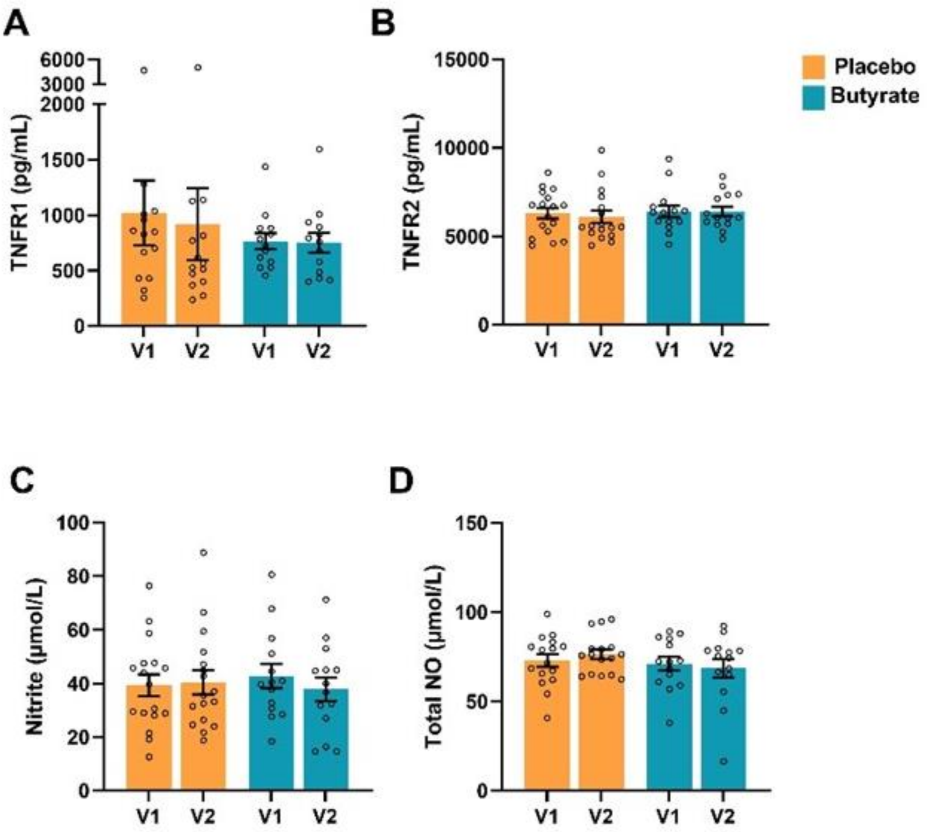

Supplemental Figure S1. A) TNF receptor 1 (TNFR1), (B) TNF receptor 2 (TNFR2), (C) Nitrite, and (D) total nitric oxide (NO) at baseline (V1) and the end of the study (V2). Orange bars indicate serum levels from patients who received placebo and blue bars indicate patients who received butyrate. Data are presented as mean ± SEM (N=14- 17 placebo group, N=13-16 butyrate group). .

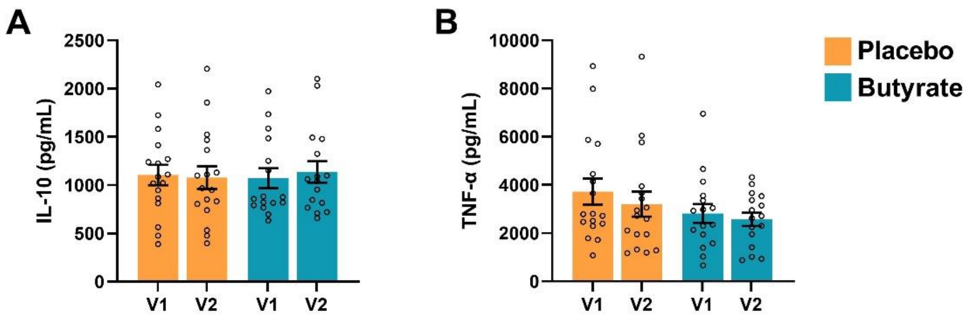

Supplemental Figure S2. ***Ex vivo*** **LPS stimulation of whole blood samples to induce IL-10** (A) and TNF-α (B) plasma concentrations. Orange bars indicate whole blood samples from patient who received placebo and blue bars indicate patients who received butyrate. Data are presented as mean ± SEM (N=17 placebo group, N=16 butyrate group). V1: Visit 1 (baseline), V2: Visit 2 (end of study).

Supplemental Table S2. ELISA data of LPS-stimulated whole blood and negative control and FACS data of restimulated PBMCs and negative control. Data are presented as mean ± SEM. Significant differences are shown as * p < 0.05, ** p < 0.01. V1: Visit 1 (baseline), V2: Visit 2 (end of study).

**Whole blood**										
	**Negative control**				**LPS-stimulated**				**LPS-stimulated**	
	**Placebo**		**Butyrate**		**Placebo**		**Butyrate**		**Placebo**	Butyrate
	Visit 1	Visit 2	Visit 1	V2	Visit 1	Visit 2	Visit 1	Visit 2	V2-V1	V2-V1
IL-10 (pg/mL)	6.245 (±1.321)	4.024** (±0.913)	8.526 (±1.891)	3.992* (±0.741)	1104.4 (±105.60)	1076.8 (±117.71)	1071.0 (±102.91)	1136.4 (±111.18)	-27.65 (±64.72)	65.46 (±102.2)
TNF-α (pg/mL)	21.43 (±6.708)	19.32** (±6.771)	24.764 (±6.951)	21.976* (±6.796)	3724.9 (±538.58)	3206.0 (±518.69)	2814.1 (±388.74)	2571.5 (±276.45)	-518.9 (±258.6)	-242.7 (±239.6)
PBMC										
	**Negative control**				**Restimulated**				**Restimulated**	
	Placebo		Butyrate		Placebo		Butyrate		Placebo	Butyrate
	Visit 1	Visit 2	Visit 1	Visit 2	Visit 1	Visit 2	Visit 1	Visit 2	V2-V1	V2-V1
IL-10+ out of CD14+ (%)	0.55 (±0.18)	0.30 (±0.10)	0.22 (±0.04)	0.34 (±0.14)	0.46 (±0.11)	0.40 (±0.17)	0.41 (±0.14)	0.63 (±0.21)	-0.05 (±0.10)	0.22 (±0.26)
TNF-α+ out of CD14+ (%)	0.40 (±0.13)	0.10* (±0.03)	0.25 (±0.06)	0.17 (±0.06)	0.54 (±0.08)	0.39 (±0.07)	0.68 (±0.23)	0.83 (±0.25)	-0.16 (±0.09)	0.14 (±0.15)
TLR4+ out of CD14+ (%)	38.57 (±3.85)	38.86 (±4.71)	35.79 (±2.85)	33.81 (±3.92)	28.29 (±2.22)	28.41 (±3.22)	34.01 (±3.49)	33.83 (±3.11)	0.12 (±2.13)	-0.18 (±2.63)
TLR4 (MFI)	2509.0 (±109.5)	2414.6* (±124.6)	2485.3 (±136.9)	2449.1 (±140.3)	2288.4 (±98.07)	2239.3 (±107.7)	2402.6 (±100.9)	2372.9 (±110.6)	-49.03 (±23.49)	-26.69 (±44.45)

Supplemental Table S3. FACS and ELISA data of LPS-stimulated, αCD3/CD28-stimulated, αCD3/CD28-stimulated+restimulated PBMCs and negative control. Data are presented as mean ± SEM. Significant differences are shown as * p < 0.05, ** p < 0.01, *** p < 0.001. V1: Visit 1 (baseline), V2: Visit 2 (end of study).

PBMC (FACS)														
	**Negative control**				**LPS-stimulated**				**αCD3/CD28-stimulated**				**αCD3/CD28-stimulated**	
	Placebo		Butyrate		Placebo		Butyrate		Placebo		Butyrate		Placebo	Butyrate
	Visit 1	Visit 2	Visit 1	Visit 2	Visit 1	Visit 2	Visit 1	Visit 2	Visit 1	Visit 2	Visit 1	Visit 2	V2-V1	V2-V1
CD25+FoxP3+ out of CD4+ (%)	0.78 (±0.11)	0.64 (±0.09)	1.14 (±0.20)	1.03 (±0.23)	1.45 (±0.16)	1.22* (±0.11)	1.63 (±0.23)	1.59 (±0.22)	6.99 (±0.56)	6.04* (±0.43)	8.36 (±0.85)	7.53** (±0.75)	-0.95 (±0.41)	-0.83 (±0.26)
CD25+FoxP3- out of CD4+ (%)	1.23 (±0.27)	1.05 (±0.15)	1.43 (±0.33)	1.17** (±0.26)	2.65 (±0.44)	2.42 (±0.37)	2.76 (±0.48)	2.81 (±0.53)	50.06 (±3.41)	46.64 (±3.98)	51.64 (±3.70)	44.36* (±3.92)	-3.42 (±2.07)	-7.28 (±2.97)
CD25+ out of CD4+ (%)	1.63 (±0.23)	1.37 (±0.15)	2.29 (±0.48)	1.91* (±0.44)	2.93 (±0.18)	2.65 (±0.19)	3.50 (±0.50)	3.59 (±0.50)	56.20 (±3.40)	51.65 (±3.89)	59.34 (±3.85)	51.04* (±4.03)	-4.55 (±2.27)	-8.30 (±3.11)
RORγ+ out of CD4+ (%)	0.46 (±0.10)	0.73 (±0.45)	0.43 (±0.07)	0.32* (±0.05)	0.49 (±0.10)	0.33* (±0.06)	0.43 (±0.07)	0.36 (±0.07)	6.37 (±1.31)	4.94* (±1.01)	7.34 (±1.60)	4.75** (±0.94)	-1.43 (±0.52)	-2.59 (±1.05)
Th17/Treg	2.22 (±1.50)	1.68 (±1.11)	0.62 (±0.19)	0.46 (±0.10)	0.41 (±0.12)	0.29 (±0.05)	0.35 (±0.08)	0.26 (±0.06)	0.92 (±0.16)	0.82 (±0.15)	0.86 (±0.16)	0.62 (±0.11)	-0.10 (±0.07)	-0.24 (±0.09)

PBMC (FACS)														
	**Negative control**				**LPS-stimulated**				**αCD3/CD28-stimulated**				**αCD3/CD28-stimulated**	
	Placebo		Butyrate		Placebo		Butyrate		Placebo		Butyrate		Placebo	Butyrate
	Visit 1	Visit 2	Visit 1	Visit 2	Visit 1	Visit 2	Visit 1	Visit 2	Visit 1	Visit 2	Visit 1	Visit 2	V2-V1	V2-V1
CD69+ out of CD4+ (%)	0.41 (±0.05)	0.36 (±0.05)	0.65 (±0.17)	0.41 (±0.06)	3.91 (±0.63)	3.54 (±0.56)	3.63 (±0.44)	2.99 (±0.30)	71.60 (3.55)	67.45 (±4.09)	76.10 (±2.60)	66.35* (±3.83)	-4.16 (±2.42)	-9.76 (±3.41)
CD69+CXCR3+ out of	0.19 (±0.02)	0.18 (±0.02)	0.32 (±0.11)	0.17 (±0.02)	0.65 (±0.09)	0.63 (±0.11)	0.56 (±0.05)	0.58 (±0.07)	20.11 (±1.35)	17.00** (±1.17)	20.51 (±1.88)	16.01** (±1.24)	-3.11 (±0.88)	-4.50 (±1.35)

CD4+ (%)+														
CD69+CCR6+ out of CD4+CXCR3- (%)	0.04 (±0.00)	0.03 (±0.00)	0.05 (±0.01)	0.04 (±0.01)	0.47 (±0.09)	0.43 (±0.08)	0.36 (±0.06)	0.34 (±0.05)	3.46 (±0.35)	2.83** (±0.31)	4.62 (±0.79)	3,26** (±0.46)	-0.63 (±0.20)	-1.37 (±0.55)
CCR6+ out of CD4+CXCR3- (%)	4.45 (±0.45)	3.69*** (±0.38)	4.19 (±0.55)	3.56 (±0.46)	5.69 (±0.64)	5.23 (±0.72)	4.69 (±0.60)	4.18 (±0.60)	4.57 (±0.38)	3.83** (±0.36)	5.59 (±0.80)	4.23* (±0.54)	-0.73 (±0.21)	-1.36 (±0.56)
PBMC (FACS)														
	**Negative control**				**Restimulated**				**αCD3/CD28-stimulated + restimulated**				**αCD3/CD28-stimulated + restimulated**	
	Placebo		Butyrate		Placebo		Butyrate		Placebo		Butyrate		Placebo	Butyrate
	Visit 1	Visit 2	Visit 1	Visit 2	Visit 1	Visit 2	Visit 1	Visit 2	Visit 1	Visit 2	Visit 1	Visit 2	V2-V1	V2-V1
IL-17a+ out of CD4+ (%)	0.13 (±0.03)	0.23 (±0.08)	0.15 (±0.04)	0.31** (±0.11)	0.32 (±0.05)	0.34 (±0.07)	0.48 (±0.11)	0.44 (±0.09)	0.39 (±0.08)	0.39 (±0.08)	0.67 (±0.13)	0.59 (±0.13)	0.002 (±0.03)	-0.08 (±0.08)
IFN-γ+ out of CD4+ (%)	0.12 (±0.03)	0.17 (±0.05)	0.11 (±0.03)	0.18 (±0.07)	5.28 (±1.38)	4.31* (±1.10)	5.10 (±1.49)	4.26 (±1.58)	9.02 (±1.34)	7.74** (±1.26)	10.38 (±1.73)	7.98* (±1.46)	-2.40 (±1.29)	-1.28 (±0.42)
IL-9+ out of CD4+ (%)	0.11 (±0.04)	0.20 (±0.08)	0.12 (±0.04)	0.26* (±0.11)	0.13 (±0.05)	0.19 (±0.08)	0.19 (±0.09)	0.24 (±0.08)	0.62 (±0.11)	0.53 (±0.10)	0.90 (±0.21)	0.67* (±0.16)	-0.23 (±0.11)	-0.09 (±0.05)
IL-10+ out of CD4+ (%)	0.17 (±0.04)	0.32** (0.08)	0.21 (±0.04)	0.39*** (±0.11)	0.24 (±0.06)	0.29 (±0.08)	0.29 (±0.09)	0.42 (±0.13)	0.70 (±0.11)	0.62 (±0.10)	1.18 (±0.19)	0.90 (±0.23)	-0.28 (±0.28)	-0.08* (±0.06)
TNF-α+ out of CD4+ (%)	0.24 (±0.05)	0.38** (±0.09)	0.31 (±0.06)	0.45 (±0.12)	3.37 (±0.96)	2.86 (±0.86)	3.86 (±1.31)	3.38 (±1.24)	7.92 (±1.18)	6.98*** (±1.12)	8.82 (±1.31)	6.98* (±1.23)	-1.84 (±0.75)	-0.93 (±0.23)
PBMC (ELISA)														
	**Negative control**				**LPS-stimulated**				**αCD3/CD28-stimulated**				**αCD3/CD28-stimulated**	
	Placebo		Butyrate		Placebo		Butyrate		Placebo		Butyrate		Placebo	Butyrate

	Visit 1	Visit 2	Visit 1	Visit 2	Visit 1	Visit 2	Visit 1	Visit 2	Visit 1	Visit 2	Visit 1	Visit 2	V2-V1	V2-V1
IL-17a (pg/mL)	1.80 (±0.15)	1.98 (±0.26)	2.19 (±0.15)	1.89 (±0.22)	32.34 (±5.88)	29.11 (±5.22)	40.19 (±7.50)	32.45 (±6.56)	1.78 (±0.10)	2.08 (±0.42)	1.60 (±0.17)	3.55 (±1.04)	-3.230 (±4.061)	-7.745 (±6.199)
IFN-γ (pg/mL)	2.74 (±0.51)	2.59 (±0.41)	3.17 (±0.73)	2.63 (±0.51)	638.55 (±86.95)	572.32 (±73.10)	470.00 (±60.35)	573.93 (±147.44)	140.72 (±40.02)	143.38 (±28.86)	76.83 (±18.29)	77.66 (±11.68)	-66.23 (±55.14)	-103.9 (±108.1)
IL-9 (pg/mL)	0.39 (±0.00)	0.39 (±0.00)	0.50 (±0.11)	0.39 (±0.00)	27.05 (±6.63)	23.60 (±5.25)	22.42 (±4.56)	20.73 (±5.07)	0.39 (±0.00)	0.39 (±0.00)	0.41 (±0.02)	0.50 (±0.08)	-3.443 (±3.176)	-1.684 (±3.473)
IL-10 (pg/mL)	1.17 (±0.00)	1.28 (±0.11)	1.17 (±0.00)	1.17 (±0.00)	665.61 (±178.03)	513.59 (±107.31)	492.42 (±90.63)	414.99 (±71.79)	1126.8 (±303.70)	859.77 (±117.79)	784.93 (±109.29)	918.68 (±221.55)	-152.0 (±93.16)	-77.44 (±90.58)
TNF-α (pg/mL)	3.40 (±1.41)	3.96 (±2.10)	2.96 (±0.70)	1.95 (±0.00)	1104.4 (± 152.18)	844.59 (±151.62)*	1058.6 (±163.36)	966.73 (±159.06)	2031.14 (±310.28)	1855.66 (±279.22)	1664.44 (±278.88)	1732.84 (±194.78)	-259.8 (±113.6)	-91.85 (±190.2)
IL-6 (pg/mL)	26.89 (±8.67)	22.67 (±9.71)	62.70 (±20.95)	36.76 (±11.22)	344.05 (±111.92)	277.16 (±73.36)	252.04 (±65.15)	280.25 (±130.42)	15118.2 (±1230.5)	15497.2 (±1379.5)	16167.1 (±1750.4)	13218.1 (±1121.2)	-66.88 (±77.83)	28.20 (±110.2)

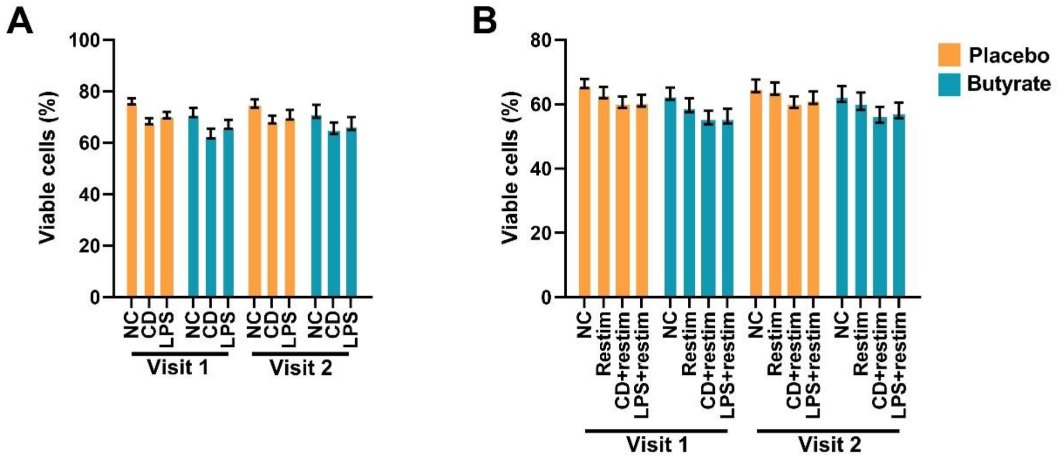

Supplemental Figure S3. Viability of PBMCs after stimulation with either LPS or αCD3/CD28 with and without restimulation (PMA and Ionomycine in presence of golgiplug) and the negative control. Data are presented as mean ± SEM (N=17 placebo group, N=15 butyrate group). CD: αCD3/CD28, NC: Negative control, Restim: restimulation.

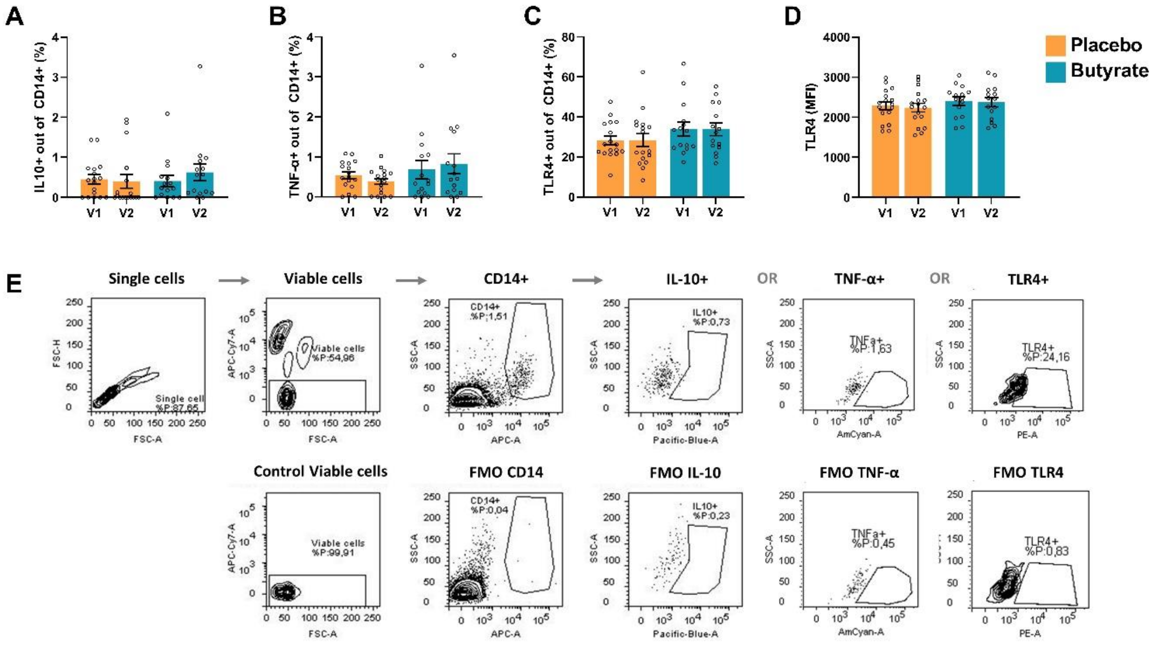

Supplemental Figure S4. FACS analysis of monocytes within restimulated PBMCs. (A) percentage of IL-10+ cells out of CD14+ cells, (B) percentage of TNF-α+ cells out of CD14+ cells, (C) percentage of TLR4+ cells out of CD14+ cells, (D) Mean fluorescence intensity (MFI) of TLR4 and (E) the used gating strategy with corresponding fluorescence minus one (FMO) controls for a representative sample. Orange bars indicate whole blood samples from patient who received placebo and blue bars indicate patients who received butyrate. Data are presented as mean ± SEM (N=17 placebo group, N=15 butyrate group). V1: Visit 1 (baseline), V2: Visit 2 (end of study).

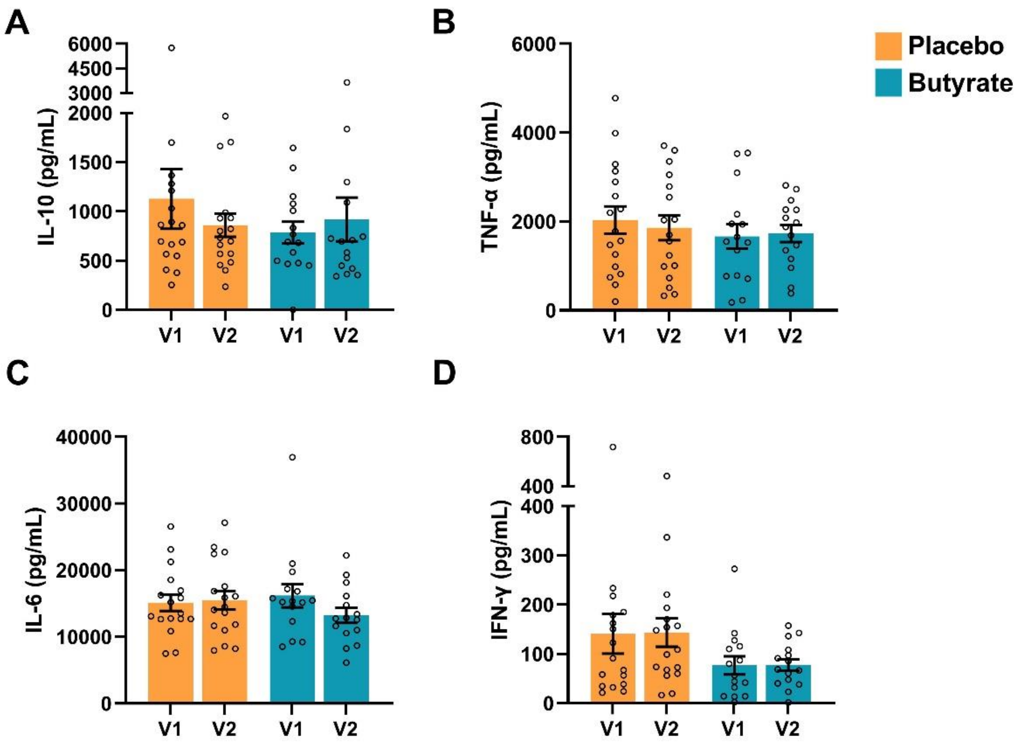

Supplemental Figure S5 IL-10 (A), TNF-α (B), IL-6 (C) and IFN-γ (D) release of LPS-stimulated PBMCs measured with ELISA. Orange bars indicate whole blood samples from patient who received placebo and blue bars indicate patients who received butyrate. Data are presented as mean ± SEM (N=17 placebo group, N=15 butyrate group). V1: Visit 1 (baseline), V2: Visit 2 (end of study).
